# Sevoflurane Postconditioning Reduces Hypoxia/Reoxygenation Injury in Cardiomyocytes via Upregulation of Heat Shock Protein 70

**DOI:** 10.4014/jmb.2103.03040

**Published:** 2021-06-11

**Authors:** Jun Zhang, Haiyan Wang, Xizhi Sun

**Affiliations:** Department of Anesthesiology, The Affiliated Yantai Yuhuangding Hospital of Qingdao University, Yantai 264000, Shandong, P.R. China

**Keywords:** Sevoflurane, hypoxia/reoxygenation injury, postconditioning, cardiomyocytes, HSP70

## Abstract

Sevoflurane postconditioning (SPostC) has been proved effective in cardioprotection against myocardial ischemia/reperfusion injury. It was also reported that heat shock protein 70 (HSP70) could be induced by sevoflurane, which played a crucial role in hypoxic/reoxygenation (HR) injury of cardiomyocytes. However, the mechanism by which sevoflurane protects cardiomyocytes via HSP70 is still not understood. Here, we aimed to investigate the related mechanisms of SPostC inducing HSP70 expression to reduce the HR injury of cardiomyocytes. After the HR cardiomyocytes model was established, the cells transfected with siRNA for HSP70 (siHSP70) or not were treated with sevoflurane during reoxygenation. The lactate dehydrogenase (LDH) level was detected by colorimetry while cell viability and apoptosis were detected by MTT and flow cytometry. Reverse transcription-quantitative polymerase chain reaction (RT-qPCR) and Western blotting were used to detect HSP70, apoptosis-, cell cycle-associated factors, iNOS, and Cox-2 expressions. Enzyme-linked immuno sorbent assay (ELISA) was used to measure malondialdehyde (MDA) and superoxide dismutase (SOD). SPostC decreased apoptosis, cell injury, oxidative stress and inflammation and increased viability of HR-induced cardiomyocytes. In addition, SPostC downregulated Bax and cleaved caspase-3 levels, while SPostC upregulated Bcl-2, CDK-4, Cyclin D1, and HSP70 levels. SiHSP70 had the opposite effect that SPostC had on HR-induced cardiomyocytes. Moreover, siHSP70 further reversed the effect of SPostC on apoptosis, cell injury, oxidative stress, inflammation, viability and the expressions of HSP70, apoptosis-, and cell cycle-associated factors in HR-induced cardiomyocytes. In conclusion, this study demonstrates that SPostC can reduce the HR injury of cardiomyocytes by inducing HSP70 expression.

## Introduction

Ischemic heart disease including myocardial infarction is the major cause of death in the world [1]. It causes blockage of the patient’s coronary circulation and myocardial blood insufficiency [2]. The definitive treatment of ischemic heart disease is ischemia/reperfusion (IR), but previous study has shown that reperfusion can initiate both transient and lethal injury, such as systemic inflammatory response, following ischemia [3]. Thus, IR injury of the heart is considered to be an important issue that is of concern to researchers. During the IR process, the cardiomyocytes suffer from hypoxia/reoxygenation (HR) which is the important mechanism of IR injury [4]. Therefore, to resist IR injury effectively, the treatment of cells injured by HR has been the focus of attention in recent years.

Clinically, to attain protection against IR injury, anesthetic is given before ischemia to induce a cardioprotective effect [5]. Sevoflurane, an ether inhalation general anesthetic agent, has low solubility in blood with a distribution coefficient of 0.6 [6]. Due to its advantages of rapid and smooth induction, quick recovery from anesthesia, low level of respiratory irritation and slight inhibition of circulation, it has been widely used in clinical anesthesia [7]. In 2009, the American College of Cardiologists recommended sevoflurane as a general volatile anesthetic agent for patients at risk for perioperative myocardial infarction during non-cardiac surgery [8]. Many clinical and experimental studies have confirmed that sevoflurane has protective profiles against IR injury of healthy cardiomyocytes [9-11]. Postconditioning of sevoflurane can be implemented at the time of reperfusion and does not have to predict an ischemic episode, so it could have significant clinical applications for patients with continuing myocardial ischemia [12, 13]. However, the underlying mechanism responsible for such cardioprotection induced by sevoflurane postconditioning (SPostC) remains largely unclear.

Some scholars have found that sevoflurane pretreatment can induce high expression of heat shock protein 70 (HSP70) in neonatal rat cardiomyocytes. However, this study did not investigate the mechanism of the effect of this regulation on myocardial injury. We aimed to further analyze the specific mechanism by which sevoflurane treatment reduces hypoxic and reoxygenation injury of cardiomyocytes through HSP70 [14]. HSP70 is a stress-inducible protein that is induced in response to a variety of stimuli including heat, ischemia, and metabolic inhibition [15, 16]. HSP70 has been strongly implicated in cardioprotection against reversible and irreversible ischemic injury [17-19]. Previous studies have demonstrated that HSP70 could suppress reactive oxygen species (ROS) generation [20], inhibit cell apoptosis [21], and attenuate calcium overload [22] to contribute to cardioprotection against HR injury. In addition, Kitahata et al. [23] reported a breakthrough finding whereby they verified the interaction between induction of HSP70 by geranylgeranylacetone (GGA) and preconditioning by sevoflurane at first, but the authors did not probe deeply into the mechanisms for these novel findings. It is still unclear whether sevoflurane protects cardiomyocytes via HSP70.

Therefore, this study intends to verify the relative contributions of SPostC to induce the expression of HSP70 to mitigate the HR injury of myocardial cells.

## Materials and Methods

### Cell Culture

The H9c2 rat cardiomyocyte line (CRL-1446, American Type Culture Collection, USA) was cultured in DMEM (P002, China) supplemented with 10% (v/v) fetal bovine serum (C0256, Beyotime, China). The cells were routinely maintained in a humid atmosphere with 5% CO_2_ at 37°C. Every 1-2 days, the culture medium was replaced. H9c2 cells were at 80-90% confluence which could be subcultured or used for experimental procedures.

### HR Cell Model Establishment and Grouping

First, the H9c2 cells were randomly divided into three groups, namely the Control group, HR group, and SPostC group. In the Control group, the H9c2 cells were cultured in 37°C, 5% CO_2_ incubator (51030966, Thermo Scientific, USA). In the HR group, the H9c2 cells were cultured in DMEM without FBS and placed into a hypoxic chamber (Thermo Fisher) containing 95% N_2_, 5% CO_2_ and 1% O_2_ for 3 h, and then returned to normoxic conditions (95% O_2_, 5% CO_2_) for 6 h to achieve reoxygenation [24, 25]. In the SPostC group, the H9c2 cells were treated with SPostC, which was performed by the following procedure: the cells were hypoxic cultured (95% N_2_, 5% CO_2_ and 1% O_2_) for 3 h, followed by reoxygenation (95% O_2_, 5% CO_2_) for 6 h; meanwhile, the H9c2 cells were exposed in a Vapor 2000 Sevoflurane Vaporizer (Draeger, Germany) containing 2.4% and 97.6% O_2_ sevoflurane for the first 20 min of reoxygenation. Next, the H9c2 cells were randomly separated into six groups, namely Control group, HR group, SPostC group, small interfering RNA targeting negative control (siNC) group, siHSP70 group, and SPostC + siHSP70 group. In the siNC group, cells were transfected with siNC vector, and then treated as in the HR group. In the siHSP70 group, cells were transfected with siHSP70 vector, and then treated as in the HR group. In the SPostC + siHSP70 group, cells were transfected with siHSP70 vector, and then treated as in the SPostC group.

### Transfection

The siRNA for HSP70 (siHSP70: 5’-GGUGGAGAUCAUCGCCAAC-3’) and negative control for siHSP70 (siNC: 5’-TTCTCCGAACGTGTCACGT-3’) were synthesized by GeneChem (China) and used for the transfection of the H9c2 cells. The transfection procedure was as follows: normal or HR-cultured or SPostC-treated H9c2 cells (2 × 10^5^) were placed into a 6-well plate and cultured until the confluence reached 80%, and then, 2 μg siHSP70 or siNC was transfected into the H9c2 cells through 3 μl Lipofectamine 3000 (L3000001, Thermo Fisher). After 48 h, the cells were harvested for later use.

### Colorimetry

A lactate dehydrogenase (LDH) colorimetric assay kit (A020-1-2, China) was used to measure LDH level. Treated H9c2 cells were incubated with substrate buffer, coenzyme І for 15 min at 37°C following instructions, and then reacted with 2, 4-dinitrophenylhydrazine for another 15 min at 37°C. Then H9c2 cells were treated with NaOH solution for 3 min at 37°C. LDH activity was measured by a microplate reader (Fluoroskan Ascent, Thermo Fisher) to read absorbance at 440 nm.

### MTT Assay

Treated H9c2 cell vitality was evaluated by MTT Cell Proliferation and Cytotoxicity Assay Kit (C0009, Beyotime). Briefly, the H9c2 cells (2 × 10^4^) were collected and 10 μl MTT solution was added to each well, then incubated for 4 h at 37°C. After that, 100 μl formazan was added to each well to solubilize the blue formazan crystals. The absorbance of the crystals was read at 570 nm by a microplate reader.

### Flow Cytometry (FCM)

Treated H9c2 cells (2 × 10^4^) were collected into a flow tube (BD352052, Becton-Dickinson, USA) and washed twice with PBS (C0221A, Beyotime) and then measured through an Annexin V-FITC/Propidine Iodide (PI) Apoptosis Detection Kit (C1062M, Beyotime). As described by the manufacturer's instructions, H9c2 cells were incubated with 195 μl Annexin V-FITC binding buffer and 5 μl Annexin V-FITC buffer, and 10 μl PI in dark for 15 min at 37°C. Flow cytometry (BD FACSCalibur, Becton-Dickinson, USA) was used to assess cardiomyocytes apoptosis. The data were analyzed by FlowJo software (VX10, Tree Star, USA).

### Enzyme-Linked Immunosorbent Assay (ELISA)

Treated H9c2 cells (2 × 10^4^) were collected to measure malondialdehyde (MDA) and superoxide dismutase (SOD) by MDA assay kit (A003-1-2, China) and SOD assay kit (A001-1-2, China), respectively. The detection processes were in accordance with the instructions. The steps for detecting MDA were as follows: 0.2 ml cardiomyocytes culture supernatant was mixed with 0.2 ml Reagent 1 at first, and then further mixed with 3 ml Reagent 2 and 1 ml Reagent 3. After centrifugation for 10 min at 3,500 ×*g*, the supernatant was collected. The red color of the supernatant, which resulted from the reaction, was read at 532 nm with a microplate reader to determine the MDA concentration. For SOD level detection, 0.05 ml cardiomyocytes culture supernatant was mixed and incubated with 1.0 ml Reagent 1, 0.1 ml Reagent 2, 0.1 ml Reagent 3 and 0.1 ml Reagent 4 for 40 min at 37°C, and then incubated with 2 ml chromogenic reagent for 10 min at 37°C. The superoxide ions reacted with 2-(4-iodophenyl)-3-(4-nitrophenol-5-phenlyltetrazolium chloride) to form a red formazan dye, which could be determined by a microplate reader at 550 nm.

### Reverse Transcription-Quantitative Polymerase Chain Reaction (RT-qPCR)

TRIzol reagent (15596026, Invitrogen, USA) was used to isolate total RNA from H9c2 cells. The cells were then incubated with reagent (0.3 ml of 100 % ethanol was added in per 1 ml of TRIzol reagent) for 2 min, and then RNA was precipitated by centrifugation for 10 min at 4°C, 12,000 ×*g*. After washing, RNA was dissolved by DEPC-treated water (750024, Thermo Fisher Scientific) and stored at 4°C. A PrimeScript RT Reagent Kit (RR037A, Takara, Tokyo, Japan) was used to prepare the cDNA templates. RNA was mixed with the 5×PrimeScript Buffer, PrimeScript RT Enzyme Mix I, RT Primer Mix, and RNase Free dH_2_O for reverse transcription. The PowerUp SYBR Green Master Mix (A25742, Thermo Fisher Scientific) was used for subsequent PCR amplification. In brief, 5 μl 2×PowerUp SYBR Green Master Mix, 1 μl appropriate primer and 1 μl cDNA, DEPC-treated water were added to make a total volume of 10 μl. All primer sequences are listed in Table 1. The reaction mixture was added to the PCR instrument (QuantStudio 3, Thermo Fisher), and GAPDH was employed for internal reference. Calculation and quantification of gene expression were based on the 2^−ΔΔCt^ method [26].

### Western Blot Analysis

Proteins in H9c2 cells were extracted by lysis buffer (P0013J, Beyotime). After determining the concentration with a BCA detection kit (P0012, Beyotime), the proteins were loaded and electrophoresed on 10% SDS polyacrylamide gel (P0012AC, Beyotime), and then transferred onto PVDF membranes (FFP26, Beyotime,). After using 5% non-fat milk to block membranes for 60 min at 37°C, the following primary antibodies were incubated with membranes overnight at 4°C: Bcl-2 (ab59348, 1:1000, 26 kDa, Abcam, USA), Bax (ab32503, 1:1000, 21 kDa, Abcam), Cleaved caspase-3 (ab49822, 1:500, 17 kDa, Abcam), iNOS (ab3523, 1:200, 135 kDa, Abcam), Cox-2 (ab15191, 1:250, 69 kDa, Abcam), CDK4 (ab199728, 1:2000, 34 kDa, Abcam), Cyclin D1 (ab16663, 1:25, 36 kDa, Abcam), HSP70 (ab181606, 1:1000, 70 kDa, Abcam), and GAPDH (ab181602, 1:10000, 36 kDa, Abcam). Following extensive washing, protein bands were incubated with the secondary antibody: Goat Anti-Rabbit IgG H&L (HRP) (ab205718, 1:2000, 42 kDa, Abcam) at 37°C for 2 h. The detection of signal was performed according to a standard ECL method (27), and analysis software (Image J 1.5i, National Institutes of Health, USA) was used for images to measured protein expression. GAPDH was used as housekeeping gene.

### Statistical Analysis

All values are reported as mean ± SD. One-way ANOVA test was used to evaluate the main treatment effect (GraphPad Prism 8, USA), followed by Tukey’s post hoc test. *p* < 0.05 was considered to indicate a statistically significant difference.

## Results

### The Effect of SPostC on LDH, MDA, SOD Levels, Cell Viability, and Apoptosis in HR-Induced H9c2 Cells

**Fig. 1:**
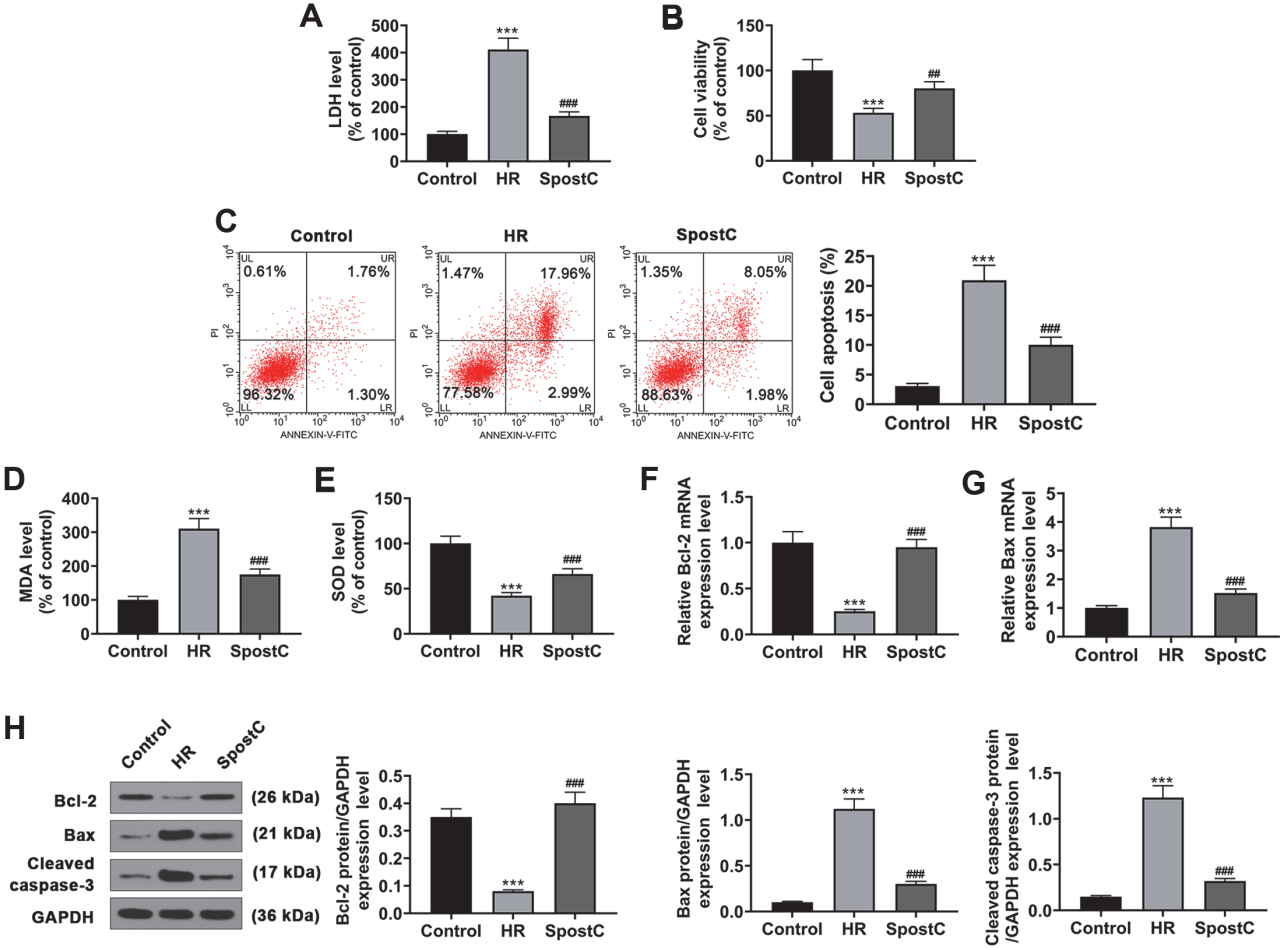
The effect of SPostC on LDH, MDA, SOD levels, cell viability, and apoptosis in HR-induced H9c2 cells. (**A**) The level of LDH in H9c2 cells that were treated with HR or SPostC and assessed by colorimetry. (**B**) Cell viability of H9c2 cells with HR or SPostC treatment and assessed by MTT assay. (**C**) Apoptosis of H9c2 cells with HR or SPostC treatment was assessed by flow cytometry. (**D-E**) MDA and SOD levels were assessed by ELISA. (**F-G**) The expression levels of Bax and Bcl-2 in the HR- or SPostC-treated cells was measured by RT-qPCR. Mean band density was normalized relative to GAPDH. (**H**) The expressions of Bax, cleaved caspase-3 and Bcl-2 were measured by Western blotting in the HR or SPostC-treated H9c2 cells. Mean band density was normalized relative to GAPDH. ***p < 0.001, vs. Control; ^###^*p* < 0.001, vs. HR. (LDH: lactate dehydrogenase; MTT: methyl thiazolyl tetrazolium; HR: hypoxic/reoxygenation; SPostC: sevoflurane postconditioning; MDA: malondialdehyde; SOD: superoxide dismutase; ELISA: enzyme-linked immunosorbent assay; RT-qPCR: reverse transcription-quantitative polymerase chain reaction).

By performing the LDH assay to observe the percentage of damaged cells, we found that compared with the cells that were cultured in normoxic conditions, the LDH level of HR-induced H9c2 cells was increased, and the level of LDH in the SPostC group was lower than that in the HR group (Fig. 1A, *p* < 0.001). As shown in Fig. 1B, the viability of H9c2 cells was decreased by HR in comparison with the Control group (*p* < 0.001), while the viability of HR-induced H9c2 cells was elevated by SPostC treatment when compared with the HR group (*p* < 0.01). Apoptosis assay also showed the similar phenomenon to LDH assay, as the apoptosis rate of H9c2 cells was improved by HR compared with the Control group, and in comparison with the HR group, the apoptosis rate of cells was decreased by SPostC treatment (Fig. 1C, *p* < 0.001). The ELISA detection showed that compared with the Control group, MDA level of the HR group was enhanced (Fig. 1D, *p* < 0.001), while SOD level was decreased (Fig. 1E, *p* < 0.001). After SPostC treatment, MDA level of HR-induced H9c2 cells was decreased and SOD level was intensified compare with the HR group (Figs. 1D-1E, *p* < 0.001). Furthermore, the transcription (Figs. 1F-1G) and translation (Fig. 1H) levels of apoptosis-related factors in H9c2 cells were evaluated. HR downregulated the level of Bcl-2 (Figs.1F and 1H) and upregulated the levels of Bax (Figs. 1G-1H) and cleaved caspase-3 (Fig. 1H) as compared with the Control group (*p* < 0.001). Meanwhile, compared with the HR group, the downregulated Bcl-2 of H9c2 cells was increased by SPostC treatment while the upregulated Bax and cleaved caspase-3 of H9c2 cells were decreased by SPostC treatment (Figs. 1F-1H, *p* < 0.001).

### The Effect of SPostC on the Expressions of Inflammatory-, Cell Cycle-Associated Factors, and HSP70 in HR-Induced H9c2 Cells

**Fig. 2:**
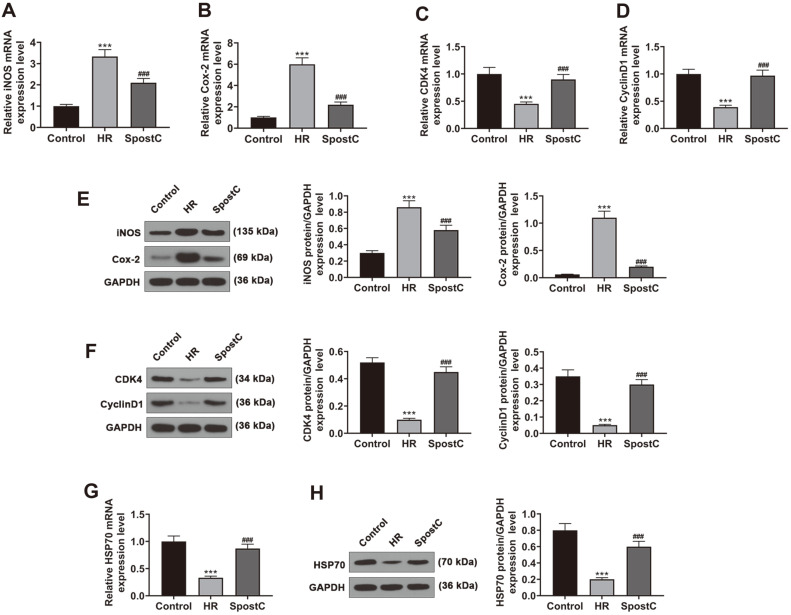
The effect of SPostC on the expressions of inflammatory-, cell cycle-associated factors, and HSP70 in HR-induced H9c2 cells. (**A-B**) The expression levels of iNOS and Cox-2 in H9c2 cells subjected to HR or SPostC were detected by RT-qPCR. Mean band density was normalized relative to GAPDH. (**C-D**) The expression levels of CDK4 and Cyclin D1 in H9c2 cells after HR or SPostC treatment were detected by RT-qPCR. Mean band density was normalized relative to GAPDH. (**E**) The expressions of iNOS and Cox-2 in H9c2 cells subjected to HR or SPostC were detected by Western blotting. Mean band density was normalized relative to GAPDH. (**F**) The expressions of CDK4 and Cyclin D1 in H9c2 cells after HR or SPostC treatment were detected by Western blotting. Mean band density was normalized relative to GAPDH. (**G**) The expression of HSP70 in H9c2 cells with HR or SPostC treatment was measured by RT-qPCR. Mean band density was normalized relative to GAPDH. (**H**) HSP70 protein expression level in H9c2 cells with HR or SPostC treatment was detected by Western blotting. Mean band density was normalized relative to GAPDH. ***p < 0.001, vs. Control; ^###^*p* < 0.001, vs. HR. (HR: hypoxic/ reoxygenation; SPostC: sevoflurane postconditioning; RT-qPCR: reverse transcription-quantitative polymerase chain reaction).

We detected the expression changes of inflammatory- related factors in H9c2 cells, and the expressions of Cox-2 and iNOS were upregulated in the HR group relative to the Control group (Figs. 2A, 2B, and 2E, *p* < 0.001). Compared with the HR group, the levels of Cox-2 and iNOS were restrained in the SPostC group (Figs. 2A, 2B, and 2E, *p* < 0.001). It was observed that cell cycle-associated factors CDK4 and Cyclin D1 levels were decreased after HR, and this decreasing trend was partially offset by SPostC (Figs. 2C, 2D, and 2F, *p* < 0.001). As shown in Fig. 2G and 2H, the expression of HSP70 in both gene and protein level of HSP70 in H9c2 cells was extremely blunted by HR than the Control group, and expression of HSP70 in HR-induced H9c2 cells was restored by SPostC (*p* < 0.001).

### SiHSP70 Reversed the Effect of SPostC on HSP70 Expression, LDH Level, Apoptosis, and Cell Viability in HR-Induced H9c2 Cells

**Fig. 3:**
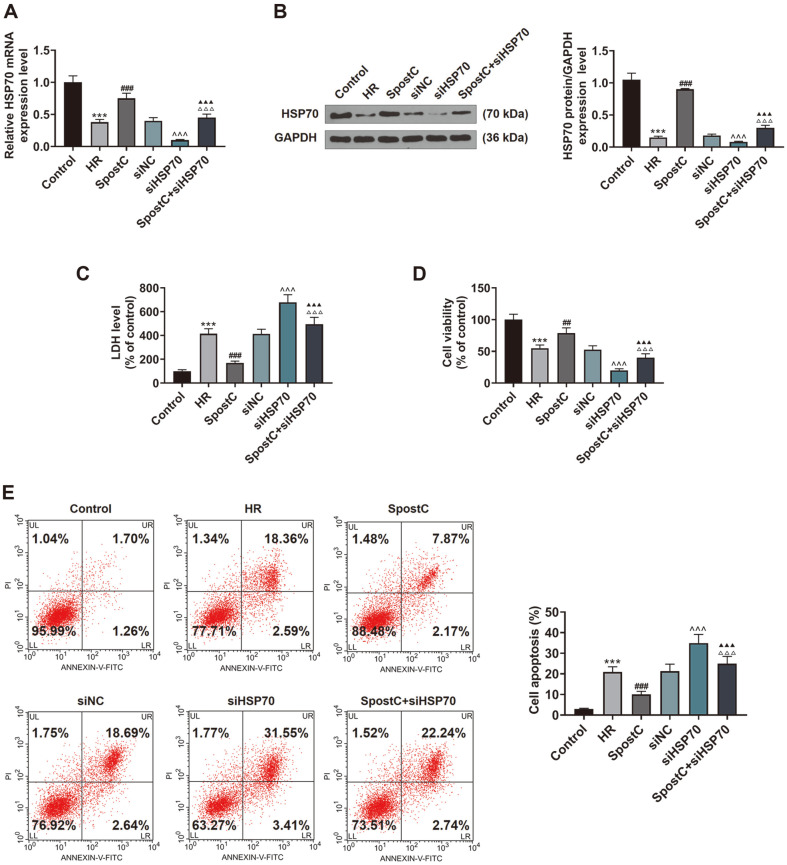
SiHSP70 reversed the effect of SPostC on HSP70 expression, LDH level, apoptosis, and cell viability in HR-induced H9c2 cells. (**A**) The level of HSP70 in H9c2 cells, which were transfected with siHSP70 and then treated with HR or SPostC, was assessed by RT-qPCR. Mean band density was normalized relative to GAPDH. (**B**) The level of HSP70 in H9c2 cells, which were transfected with siHSP70 and then treated with HR or SPostC, was assessed by Western blotting. Mean band density was normalized relative to GAPDH. (**C**) The level of LDH in H9c2 cells, which were transfected with siHSP70 and then treated with HR or SPostC, was assessed by colorimetry. (**D**) Cell viability of H9c2 cells, which were transfected with siHSP70 and then treated with HR or SPostC, was assessed by MTT assay. (**E**) Apoptosis of H9c2 cells subjected to HR injury or SPostC with low expression of HSP70 was assessed by flow cytometry. ***p < 0.001, vs. Control; ^##^*p* < 0.01, ^###^*p* < 0.001, vs. HR; ^^^*p* < 0.001, vs. siNC; ^▲▲▲^*p* < 0.001, vs. siHSP70; ^△△△^*p* < 0.001, vs. SPostC. (HR: hypoxic/reoxygenation; SPostC: sevoflurane postconditioning; HSP70: heat shock protein 70; LDH: lactate dehydrogenase; MTT: methyl thiazolyl tetrazolium; RT-qPCR: reverse transcription-quantitative polymerase chain reaction; siNC: small interfering RNA targeted negative control; siHSP70: small interfering RNA targeted HSP70).

As the data exhibited in Fig.3A-3B, siHSP70 restrained the HSP70 expression, which was reversed by co-treatment of SPostC and siHSP70 (*p* < 0.001). Furthermore, the LDH level was enhanced by siHSP70 in comparison to the siNC group, while co-treatment of SPostC and siHSP70 partially neutralized the promotion of siHSP70 on the LDH level (Fig. 3C, *p* < 0.001). And as Fig. 3D shown, siHSP70 blunted the viability of H9c2 cells, and the viability of cells in the SPostC + siHSP70 group was higher than the siHSP70 group (*p* < 0.001). The apoptosis rate of cells in the siHSP70 group was elevated more than the siNC group (Fig. 3E, *p* < 0.001). As for the SPostC + siHSP70 group, the apoptosis rate of H9c2 cells was lower than the siHSP70 group, and it was higher than the apoptosis rate of cells subjected to SPostC without siHSP70 (Fig. 3E, *p* < 0.001).

### SiHSP70 Reversed the Effect of SPostC on Oxidation-, Apoptosis-, Inflammatory-, and Cell Cycle-Associated Factors in HR-Induced H9c2 Cells

**Fig. 4:**
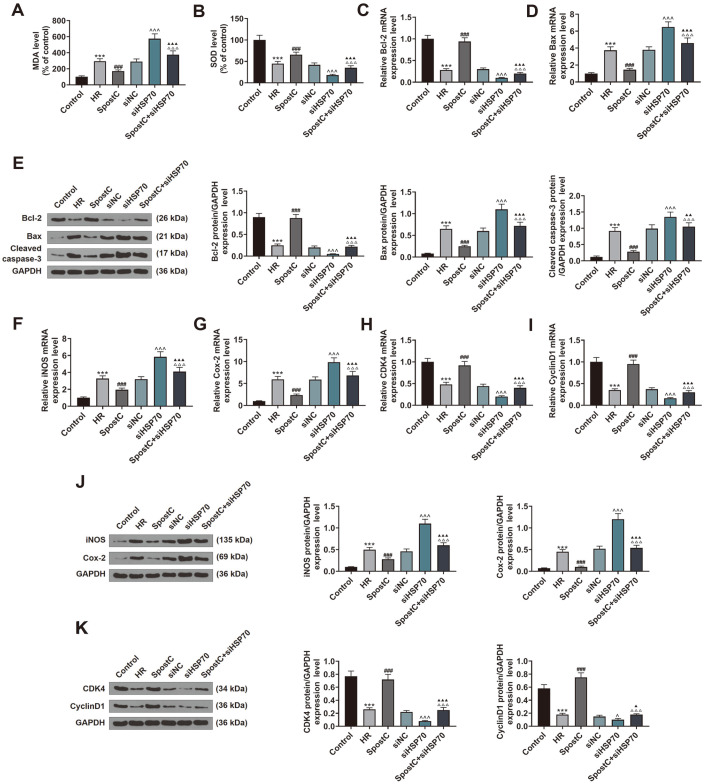
SiHSP70 reversed the effect of SPostC on oxidation-, apoptosis-, inflammatory-, and cell cycleassociated factors in HR-induced H9c2 cells. (**A-B**) MDA and SOD levels in H9c2 cells transfected with siHSP70 and treated with HR or SPostC were assessed by ELISA. (**C-D**) The expression levels of apoptosis-related genes Bax and Bcl-2 in H9c2 cells, which were transfected with siHSP70 and subjected to HR injury or SPostC treatment, were measured by RT-qPCR. Mean band density was normalized relative to GAPDH. (**E**) The expression levels of Bax, cleaved caspase-3 and Bcl-2 in H9c2 cells, which were transfected with siHSP70 and subjected to HR injury or SPostC treatment, were measured by Western blotting. Mean band density was normalized relative to GAPDH. (**F-G**) The expression levels of iNOS and Cox-2 in H9c2 cells transfected with siHSP70 and subjected to HR injury or SPostC treatment were detected by RT-qPCR. Mean band density was normalized relative to GAPDH. (**H-I**) The expression levels of CDK4 and Cyclin D1 in H9c2 cells transfected with siHSP70 and subjected to HR or SPostC treatment were detected by RT-qPCR. Mean band density was normalized relative to GAPDH. (**J**) The expressions of iNOS and Cox-2 in H9c2 cells transfected with siHSP70 and subjected to HR injury or SPostC treatment were detected by Western blotting. Mean band density was normalized relative to GAPDH. (**K**) The expressions of CDK4 and Cyclin D1 in H9c2 cells transfected with siHSP70 and subjected to HR or SPostC treatment were detected by Western blotting. Mean band density was normalized relative to GAPDH. ***p < 0.001, vs. Control; ^###^*p* < 0.001, vs. HR; ^*p* < 0.05, ^^^*p* < 0.001, vs. siNC; ^▲^*p* < 0.05, ^▲▲^*p* < 0.01, ^▲▲▲^*p* < 0.001, vs. siHSP70; ^△△△^*p* < 0.001, vs. SPostC. (MDA: malondialdehyde; SOD: superoxide dismutase; ELISA: enzyme-linked immunosorbent assay; HR: hypoxic/reoxygenation; SPostC: sevoflurane postconditioning; RT-qPCR: reverse transcription-quantitative polymerase chain reaction; siNC: small interfering RNA targeted negative control; siHSP70: small interfering RNA targeted HSP70).

Experimental results showed that siHSP70 upregulated the MDA level and downregulated the SOD level of H9c2 cells transfected with siHSP70 than the siNC group (Figs. 4A-4B, *p* < 0.001). Comparing with the siHSP70 group, the MDA level of H9c2 cells was decreased and the SOD level was increased in the SPostC+siHSP70 group (Figs. 4A-4B, *p* < 0.001). As observed in Figs. 4C-4E, Bcl-2 was downregulated, Bax and cleaved caspase-3 levels were upregulated in the siHSP70 group relative to the siNC group (*p* < 0.001). Co-treatment of SPostC and siHSP70 partially offset the regulation of SPostC/siHSP70 on Bcl-2, Bax and cleaved caspase-3 levels (Figs. 4C-4E, *p* < 0.01). We also detected inflammatory-related factor expression, the data was exhibited in Figs. 4F, 4G, and 4J, Cox-2 and iNOS expressions were upregulated with the effect of siHSP70 as compared to the siNC group (*p* < 0.001). The expressions of Cox-2 and iNOS in the SPostC+siHSP70 group were restrained in the comparison of the siHSP70 group (Figs. 4F, 4G, and 4J, *p* < 0.001), but expressions of Cox-2 and iNOS in the SPostC+siHSP70 group cells were significantly higher than in the H9c2 cells just treated with SPostC (the SPostC group, Figs. 4F, 4G, and 4J, *p* < 0.001). In addition, the expressions of CDK4 and Cyclin D1 were detected by Western blot and RT-qPCR, and the results were showed in Figs. 4H, 4I, and 4K. siHSP70 apparently alleviated the CDK4 and Cyclin D1 expressions in HR-induced cells compared with the siNC group (*p* < 0.05), while the above effect was reversed by co-treatment of SPostC and siHSP70 (*p* < 0.05).

## Discussion

Our findings suggest that low expression of HSP70 could eliminate the protective effect of SPostC on H9c2 cells injured by HR, and we concluded that SPostC might protect cardiomyocytes from HR injury by inducing HSP70 expression.

Heart disease is a common illness that can trigger cardiomyocytes hypoxia and then elicit cell proliferation, hypertrophy and death [28]. IR is one of the effective treatments to improve hypoxia and cause cardiac resuscitation, but it could lead to additional IR injury for patients [29]. Sevoflurane exerts protective effects on myocardial IR injury in clinical experiment [30, 31], thus it is suggested for use as a general volatile anesthetic agent for patients, especially those with myocardial infarction [32]. Therefore, an in-depth understanding of the protective mechanism of sevoflurane against myocardial IR injury may be of value for the clinical treatment of cardiac diseases such as cardiomyopathy. Both sevoflurane preconditioning and postconditioning have been proved to play positive roles in myocardial cardioprotection [33, 34], and there are many proposed hypotheses for the protective mechanisms although they [35-38] are not fully understood.

HR model is commonly used to mimic the injury of IR to the body or organs at the cellular level; it is simple, controllable and reproducible without other types of cell disturbance [39]. Herein, we constructed the HR model to study the protection mechanism of sevoflurane to H9c2 cells. LDH is a key enzyme in the control of energy metabolism, and it regulates the levels of lactate interconverted by pyruvate in accordance with oxygen availability [40], so that LDH levels increase in cells cultured with hypoxic compared with cells cultured in normal conditions. When cells are under oxidative stress, levels of ROS increase and cause cell injury via inactivation of antioxidant enzymes such as SOD and consumption of antioxidants [41]. In the meantime levels of the products of lipid peroxidation MDA increase with apoptosis [42]. In this study, we discovered that SPostC markedly decreased the LDH levels, apoptosis rate and MDA level, and increased SOD level of hypoxia H9c2 cells, which indicated that SPostC might effectively reduce HR injury of H9c2 cells. Expression levels of apoptosis-related factors such as Bcl-2, Bax and cleaved caspase-3 are different at various stages of apoptosis, and are used to characterize apoptosis [43]. By detecting changes in both mRNA and protein-associated apoptosis expression levels, we observed that the expression of Bcl-2 in H9c2 cells treated with SPostC after HR was significantly increased. In the meantime, Bax and cleaved caspase-3 expression levels were decreased, and those results show that SPostC decreased apoptosis of HR-damaged H9c2 cells. HR can result in elevated proinflammatory cytokine production of the injured cells, and cells’ inflammatory response can further aggravate organ damage [44]. iNOS and Cox-2 are two types of protein molecules related to inflammation, and coinduction of iNOS and Cox-2 related to inflammatory reaction has been proved by many previous studies [45]. Thus, we regard expression levels of iNOS and Cox-2 as indicators to evaluate the degree of HR injury, and we discovered that Cox-2 and iNOS downregulated. Also, cell cycle-associated factors CDK4 and Cyclin D1 are also associated with cellular activity [46], and they are expressed at low levels in HR injured cells. In our study, CDK4 and Cyclin D1 expression levels increased in HR-induced H9c2 cells after SPostC. In agreement with previous reports and clinical research [47, 48], our data suggest that SPostC plays an essential role in protecting cardiomyocytes against HR injury.

HSP70 has been shown to have cardioprotective effects [49]. Therefore, regulating the expression and function of HSP70 may be one of the important directions in the study of cardiac ischemia reperfusion [50]. Numerous studies have demonstrated that preconditioning and postconditioning are involved in the cardioprotection [51, 52]. Accumulated studies have revealed that sevoflurane can induce the expression of HSP70 [16]. However, the relationship between the effect of sevoflurane to protect cardiomyocytes and the ability of sevoflurane to induce HSP70 expression has not yet been explored. Thus, we supposed that sevoflurane protects H9c2 cells via inducing HSP70 based on the evidence mentioned above. HSP70 as a member of the HSP protein family could limit cell damage via blunting the death signal or preventing the activation or activity of sensor or effector molecules [53]. The results in this study indicated that sevoflurane upregulated the expression of HSP70 in H9c2 cells, and the protective effect of SPostC on H9c2 cells was partially reversed by siHSP70, and this mechanism was related to the regulation of viability, apoptosis, cell cycle and oxidative stress.

On the basis of this data, we can draw the following conclusion that siHSP70 blocked the cardioprotective effect of SPostC, and prove that our hypothesis is plausible.

In summary, SPostC protects cardiomyocytes against myocardial HR injury effectively, and this cardioprotective effect is mediated by upregulation of HSP70. Our research provides a new approach for exploring the mechanism of sevoflurane protecting cardiomyocytes against HR injury.

## Figures and Tables

**Table 1:** Primer sequences for RT-qPCR.

Primer name	Primer sequence (5'-3')
Bcl-2-Forward	GGACAACATCGCTCTGTGGA
Bcl-2-Reverse	AATCCACTCACACCCCAACC
Bax-Forward	GAAACCCCTGGATGTACCCC
Bax-Reverse	GCCCTATTGTGGTGGGATGG
HSP70-Forward	GCCAAACGGTTCATCGGGA
HSP70-Reverse	AGGTGCTATTACCAGCAAGGT
iNOS-Forward	AACTTCTGACAGAGGCTCCC
iNOS-Reverse	TTGCTGTTTTCACCCTGCTC
Cox-2-Forward	GACGAAATCAACAACCCCGT
Cox-2-Reverse	TATTGGCAGAACGACTCGGT
CDK4-Forward	AGCTAAATCCCCAACCCCTC
CDK4-Reverse	CTGGGTAGGCTGGGACTATG
Cyclin D1-Forward	TCTGGAAGCAATGTGTCCCT
Cyclin D1-Reverse	GCTGTCCTTTACCTCCACCT
GAPDH-Forward	CCATCTTCCAGGAGCGAGAT
GAPDH-Reverse	TGCTGATGATCTTGAGGCTG
